# Editorial: Recent 3D Tumor Models for Testing Immune-Mediated Therapies

**DOI:** 10.3389/fimmu.2021.798493

**Published:** 2021-11-18

**Authors:** Jacques Zimmer, Roberta Castriconi, Silvia Scaglione

**Affiliations:** ^1^ Department of Infection and Immunity, Luxembourg Institute of Health, Esch-sur-Alzette, Luxembourg; ^2^ Department of Experimental Medicine, University of Genova, Genova, Italy; ^3^ IEIIT Institute, National Research Council (CNR), Roma, Italy; ^4^ Department R&D, React4life s.r.l., Milan, Italy

**Keywords:** 3D tumor models, microfluidics, organ-on-chip, bioprinting, colon cancer, neuroblastoma, glioblastoma, CAR-T cells

For a long time, cancer research was based on the culture of cell lines and primary tumor cells grown in 2 dimensions (2D), as well as on animal models mainly based on the use of rodents such as mice and rats. However, *in vitro* 2D conventional cell cultures fail to accurately predict the drug responses in humans, as they do not properly resemble the spatial complexity of the human tissue microenvironment; on the other side, research on living animals did not completely meet the public agreement, pointing out ethical questions which have been addressed and regulated by the European Community. In addition to the ethical issues, the heterogeneity of housing conditions, of microbiota and chow compositions and the inability to reproduce the complex interplay between tumor cells and human microenvironment represent additional weaknesses of the most utilized *in vivo* models (1). Therefore, the progressive switch to 3D experimental material is accompanied by several advantages converging in a better reproducibility of the results among different labs.

Current 3D cultures are based on the establishment of different models including tumor organoids. These are derived from epithelial cells of many organs and can be ideally established from each patient, with the possibility to comparatively analyze tumor and normal tissue from the same individual, in the context of personalized medicine (2). As they originate from stem cells, they have the capacity to self-organize and self-renew (2). There are also several possibilities to mimic the tumor microenvironment (TME) in 3D structures. This TME contains various organic and inorganic molecules belonging to extracellular matrix and several non-cancerous cell types that nevertheless create a strongly immunosuppressive environment rendering the cancer resistant to many treatment options (3). The 3D models likewise allow to evaluate treatment efficiency for the individual patient, for example the response to checkpoint inhibitors, correlated with clinical responses (4). Experimental treatments and therapeutic combinations can be tested in 3D tumor spheroid microarrays bringing together NK92-CD16 cells and tumor cell lines with anti-tumor antibodies triggering antibody-dependent cellular cytotoxicity by the natural killer (NK) cell line (5). However, the current 3D models still have some unmet challenges, such as the absence of vascularization in the organoids, or the organ-organ cross-talk, that might be circumvented by the use of organs-on-chip technologies (6).

This Research Topic is dedicated to some recent aspects of the 3D “revolution”, describing or reviewing in details the different models. The articles likewise critically discuss the most relevant weaknesses of the 3D models, also suggesting possible methodological approaches to address and resolve them.

Thus, in the first paper, Sargenti et al. report on the relative heterogeneity of weight and size of spheroids derived from four different colon cancer cell lines, which might of course influence the data and the conclusions obtained. With the aim to *in vitro* combine 3D cancer models and fluid dynamic conditions (7), the authors describe a flow-based method focused on a quantitative analysis of weight, size and mass density of cancer spheroids, as well as a measurement of cell infiltration. They use their system to test the cytotoxic effect of NK cells on the tumor spheroids, appreciated through measurements of weight loss and size reduction. The latter are cell line-dependent and thus most likely reflect the patient-specific behavior of primary spheroids and the suitability of the model for personalized medicine and testing of immunotherapeutic modalities.

In the second manuscript, Corallo et al. review emerging 3D technologies to preclinically study neuroblastoma, an aggressive pediatric neuroectodermal tumor with a current unmet therapeutic need (8). Due to the high heterogeneity of this cancer, the development of standardized 3D systems represents a real challenge. However, some 3D models are in development or already available, thus allowing preclinical drug and immunotherapy testing similar to other types of cancer. The paper also widely discusses various properties of extracellular matrix (ECM) components (e.g. scaffolds) mostly used in 3D models and crucial for recapitulating the *in vivo* tumor microenvironment.

Boucherit et al. contributed with a general but comprehensive overview of the most utilized 3D tumor models, comparing cell lines-based structures with patient-derived cultures, 3D bioprinting and organs-on-chip approaches. In each case, advantages and potential pitfalls are discussed.

In the next manuscript, Klein et al. from the Niclou group provide a specialized critical and exhaustive review about glioblastoma (GBM) organoids. In contrast to most other tumors that are at least possible to treat, GBM almost always relapses and the patient survival (median) is less than two years even after the currently available optimized treatment (surgery, radiotherapy, temozolomide) (9). The authors suggest that most clinical trials in GBM fail due to the lack of appropriate preclinical studies mostly neglecting the *in vivo* TME and its properties. The article contains a full table indicating advantages and disadvantages of cell-based models and organoids even genetically-engineered.

Sbrana et al. focused their contribution on chronic lymphocytic leukemia (CLL) which represents the most frequent and still uncurable adult type of leukemia (10). With a 3D-bioprinting method, they achieve to develop a long-term CLL culture model able to assess growth characteristics, functional behavior of the leukemic cells and their sensitivity to potential innovative treatments.

Finally, Grunewald et al. describe another 3D-bioprinting system used to investigate chimeric antigen receptor (CAR)-T cells targeting neuroblastoma *via* the adhesion molecule L1CAM. The cell culture system was viable over time and the CAR-T cells infiltrated the 3D-bioprint, delivering a kind of proof-of-concept about the appropriateness of this preclinical model, mimicking the patient’s TME, to test the anti-tumor efficacy of a given CAR construct.

In conclusion, the Research Topic provides a complete and critic overview of the most used and reliable 3D tumor models that represent crucial tools of future cancer research whose optimization will go hand-in-hand with the increasing development of anti-tumor immunotherapeutic strategies.

## Author Contributions

JZ, RC, and SS wrote the editorial and invited authors to participate in the collection. All authors contributed to the article and approved the submitted version.

## Funding

This work was supported by the European Union’s Horizon 2020 research and innovation programme under grant agreement No 801159.

## Conflict of Interest

Author SS is employed by React4life s.r.l.

The remaining authors declare that the research was conducted in the absence of any commercial or financial relationships that could be construed as a potential conflict of interest.

## Publisher’s Note

All claims expressed in this article are solely those of the authors and do not necessarily represent those of their affiliated organizations, or those of the publisher, the editors and the reviewers. Any product that may be evaluated in this article, or claim that may be made by its manufacturer, is not guaranteed or endorsed by the publisher.
